# Longitudinal changes of spinal cord grey and white matter following spinal cord injury

**DOI:** 10.1136/jnnp-2021-326337

**Published:** 2021-07-31

**Authors:** Gergely David, Dario Pfyffer, Kevin Vallotton, Nikolai Pfender, Alan Thompson, Nikolaus Weiskopf, Siawoosh Mohammadi, Armin Curt, Patrick Freund

**Affiliations:** 1 Spinal Cord Injury Center, Balgrist University Hospital, University of Zurich, Zurich, Switzerland; 2 Department of Brain Repair and Rehabilitation, UCL Institute of Neurology, London, UK; 3 Department of Neurophysics, Max Planck Institute for Human Cognitive and Brain Sciences, Leipzig, Germany; 4 Felix Bloch Institute for Solid State Physics, Faculty of Physics and Earth Sciences, Leipzig University, Leipzig, Germany; 5 Department of Systems Neuroscience, University Medical Center Hamburg-Eppendorf, Hamburg, Germany; 6 Wellcome Trust Centre for Neuroimaging, UCL Institute of Neurology, London, UK

## Abstract

**Objectives:**

Traumatic and non-traumatic spinal cord injury produce neurodegeneration across the entire neuraxis. However, the spatiotemporal dynamics of spinal cord grey and white matter neurodegeneration above and below the injury is understudied.

**Methods:**

We acquired longitudinal data from 13 traumatic and 3 non-traumatic spinal cord injury patients (8–8 cervical and thoracic cord injuries) within 1.5 years after injury and 10 healthy controls over the same period. The protocol encompassed structural and diffusion-weighted MRI rostral (C2/C3) and caudal (lumbar enlargement) to the injury level to track tissue-specific neurodegeneration. Regression models assessed group differences in the temporal evolution of tissue-specific changes and associations with clinical outcomes.

**Results:**

At 2 months post-injury, white matter area was decreased by 8.5% and grey matter by 15.9% in the lumbar enlargement, while at C2/C3 only white matter was decreased (−9.7%). Patients had decreased cervical fractional anisotropy (FA: −11.3%) and increased radial diffusivity (+20.5%) in the dorsal column, while FA was lower in the lateral (−10.3%) and ventral columns (−9.7%) of the lumbar enlargement. White matter decreased by 0.34% and 0.35% per month at C2/C3 and lumbar enlargement, respectively, and grey matter decreased at C2/C3 by 0.70% per month.

**Conclusions:**

This study describes the spatiotemporal dynamics of tissue-specific spinal cord neurodegeneration above and below a spinal cord injury. While above the injury, grey matter atrophy lagged initially behind white matter neurodegeneration, in the lumbar enlargement these processes progressed in parallel. Tracking trajectories of tissue-specific neurodegeneration provides valuable assessment tools for monitoring recovery and treatment effects.

## Introduction

Spinal cord injury (SCI) is a devastating event that usually leads to permanent impairments.^1^ Currently, there is no cure for SCI, but rehabilitation has been shown to improve outcome.^2^ Traumatic and non-traumatic SCI cause not only focal damage to the injury site (primary injury),^3–5^ but also trigger a cascade of secondary pathological processes that propagate above and below the injury site,^1 6 7^ affecting also the brain.^8–11^ However, little is known about the spatiotemporal dynamics of remote spinal cord white and grey matter atrophy, which are key processes involved in the patients' long-term recovery and targets of interventions.^12 13^

Experimental studies revealed early anterograde and retrograde neurodegeneration of the sensory and motor tracts above and below the injury.^14 15^ In patients with chronic traumatic SCI, significant tissue-specific atrophy and altered microstructure was detected both in the cervical^16^ and lumbar spinal cord.^17^ Starting at the acute stage, these remote macrostructural (atrophy) and microstructural changes have been shown to continuously progress over the first year after injury at C2/C3 level,^8 10^ with cord atrophy decelerating after 2 years.^18^ In addition, there is evidence that the magnitude of remote cord atrophy and reduction in myelin (demyelination) is associated with clinical impairment.^8 10^ However, these studies focused only on remote degeneration rostral to the injury site by means of cross-sectional cord area, with no information on longitudinal tissue-specific changes. Based on the literature, we hypothesised that (i) grey and white matter microstructural and macrostructural changes are already present early after injury and (ii) continue to worsen over time above and below the level of injury.

## Methods

### Study participants

Thirteen patients with traumatic and three with non-traumatic SCI (three females, age (mean±SD): 50.3±16.0 years), admitted consecutively into the rehabilitation programme at the Balgrist University Hospital (Zurich, Switzerland) between January 2016 and March 2019, and ten healthy controls recruited during the same time period (three females, age: 45.3±19.2 years) participated in this 1.5-year longitudinal study (table 1). Inclusion criteria were (1) no pre-existing neurologic or mental disorders, (2) no history of head and brain lesions, (3) no MRI contraindications and (4) no pregnancy. Participants were scanned at baseline (n=14) and at 6 (n=12) and 16 months (n=12) follow-up. In patients, the mean (±SD) interval to the first scan following injury was 2.1 (±1.3) months, to the second scan 8.2 (±4.2) months and to the third scan 18.1 (±7.1) months. The baseline scans of the healthy controls served as the healthy cohort in a previous cross-sectional study.^17^

**Table 1:** Demographic, clinical, injury and lesion information of the patients with spinal cord injury

ID	Sex	Injury level	AIS	Type	Aetiology	Motor score (maximum 50)		Light touch score (maximum 112)		Pin prick score (maximum 112)		Lesion area (mm^2^)		Lesion width (mm)		Lesion length (mm)		Width of tissue bridges (mm)	
						2 mo	18 mo	2 mo	18 mo	2 mo	18 mo	2 mo	18 mo	2 mo	18 mo	2 mo	18 mo	2 mo	18 mo
1	M	C5	B	Traumatic	Locked facet fracture C4/5	11	14	48	30	42	16	127.7	144.6	19.5	22.2	9.5	9.2	0.0	0.6
2	M	T12	C	Traumatic	Dislocation fracture L2	30	41	78	72	78	72	n.a.	n.a.	n.a.	n.a.	n.a.	n.a.	n.a.	n.a.
3	F	L1	C	Traumatic	Burst fracture T12	57	96	84	96	84	96	n.a.	n.a.	n.a.	n.a.	n.a.	n.a.	n.a.	n.a.
4	M	T12	D	Traumatic	Cord contusion T12	97	100	94	105	112	94	0.0	0.0	0.0	0.0	0.0	0.0	8.5	7.2
5	M	C4	D	Traumatic	Contusion, pre-existing stenosis C5/6	81	88	63	87	104	75	60.4	6.5	19.0	5.0	3.7	1.9	1.7	4.1
6	M	C5	D	Traumatic	Hyperextension–distraction C6/7	92	100	110	112	106	105	4.8	0.0	3.0	0.0	2.6	0.0	4.9	6.8
7	M	C4	D	Traumatic	Cord contusion C3/4	98	100	111	112	112	112	0.0	0.0	0.0	0.0	0.0	0.0	7.2	7.0
8	F	T8	D	Traumatic	Fracture T9 perioperative	87	97	99	112	112	98	n.a.	n.a.	n.a.	n.a.	n.a.	n.a.	n.a.	n.a.
9	M	C4	D	Traumatic	Contusion, pre-existing stenosis C5/6	93	100	108	112	96	112	0.0	0.0	0.0	0.0	0.0	0.0	6.6	6.3
10	M	C4	D	Traumatic	Contusion, pre-existing stenosis C5/6	93	100	69	110	62	97	60.7	14.6	5.3	6.5	23.3	2.9	3.1	4.5
11	M	T9	D	Traumatic	Burst fracture T12	100	100	110	110	112	112	n.a.	n.a.	n.a.	n.a.	n.a.	n.a.	n.a.	n.a.
12	M	C7	D	Traumatic	Dislocation fracture C6/7	88	82	112	112	112	112	0.0	n.a.	0.0	n.a.	0.0	n.a.	6.2	n.a.
13	M	C2	D	Traumatic	Hyperextension–distraction C3/4	56	88	58	108	56	98	17.1	n.a.	5.1	n.a.	4.7	n.a.	3.0	n.a.
14	M	T10	A	Non-traumatic	Ischaemia T10	50	50	77	78	68	78	55.2	37.4	23.5	23.6	4.7	2.9	0.0	0.0
15	M	L1	A	Non-traumatic	Ischaemia T12	72	78	95	98	86	87	217.3	n.a.	56.8	n.a.	5.7	n.a.	0.0	n.a.
16	F	T6	D	Non-traumatic	Cavernoma T6/7	100	100	97	95	112	112	18.3	n.a.	8.8	n.a.	3.0	n.a.	3.2	n.a.

AIS, American Spinal Injury Association Impairment Scale; mo, month; n.a., not available (due to artefacts caused by orthopaedic implants).

### MRI acquisition protocol

**Figure 1:**
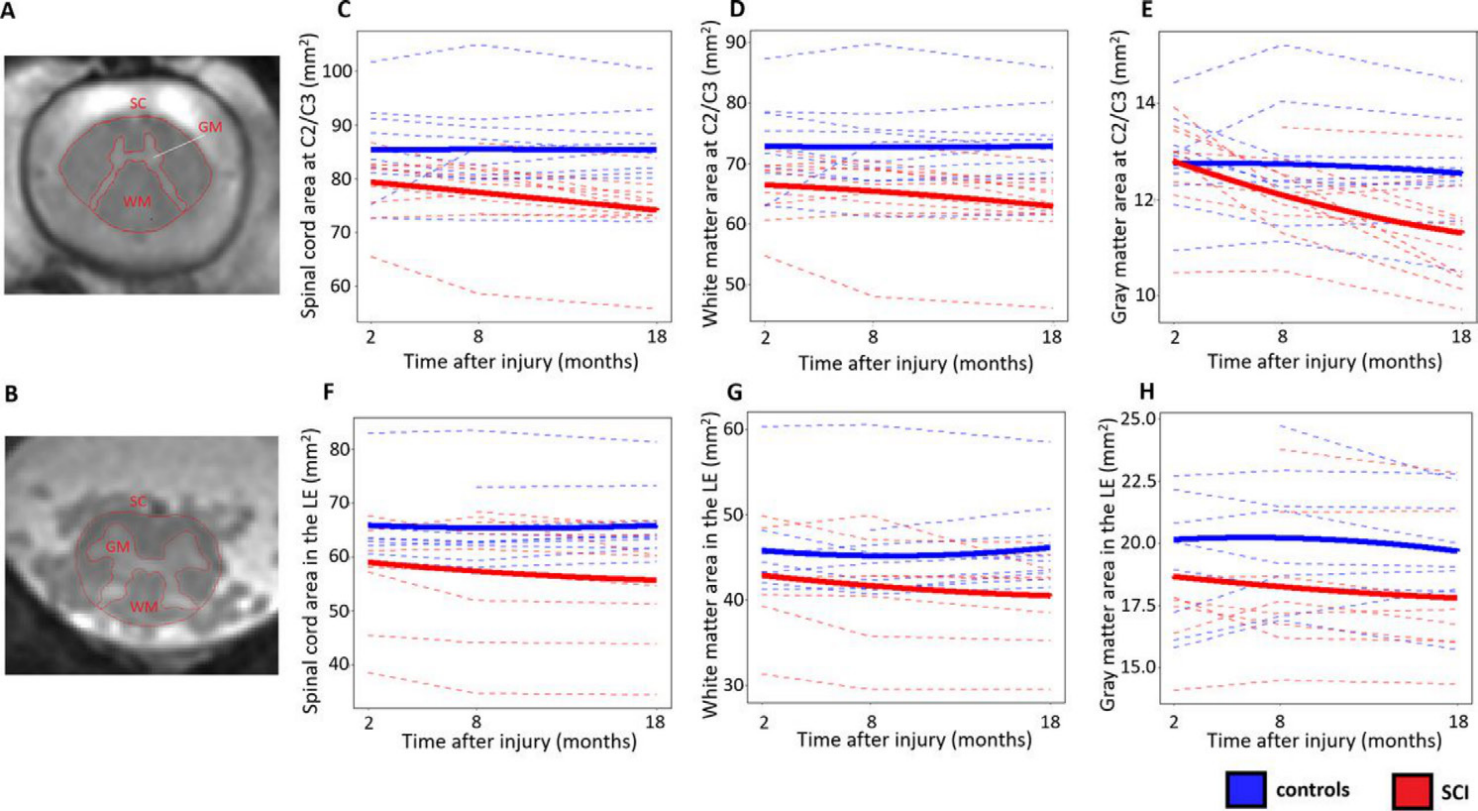
Longitudinal changes in cross-sectional areas. (A, B) Cross-sectional areas of spinal cord (SC), white matter (WM) and grey matter (GM) were measured at two locations, (A) above the injury at C2/C3 and (B) below the injury in the lumbar enlargement (LE), by means of semiautomatic and manual segmentation. (C–H) Longitudinal changes in cross-sectional areas of SC, WM and GM in controls (blue lines) and patients with spinal cord injury (SCI) (red lines), over three timepoints within the first year after injury. Dashed lines represent evolution in individual subjects, while the solid lines represent the quadratic fit in controls and patients with SCI separately.

All MRI measurements were done on a clinical 3T Siemens Skyra^Fit^ system (Erlangen, Germany), using the standard transmit coil for transmission and the 16-channel head and neck coil and standard spine matrix coil for reception. Two anatomical locations were measured: the upper cervical cord and the lumbar enlargement located between T11-L1 (figure 1A, B). To identify the location of the lumbar enlargement, a sagittal T2-weighted two-dimensional (2D) turbo spin echo sequence of the lumbar region was acquired with the following parameters: 23 slices, slice thickness=3 mm, in-plane resolution=0.6×0.6 mm^2^, field of view (FOV)=160×160 mm^2^, echo time (TE)=96 ms, repetition time (TR)=5510 ms, flip angle=150°, readout bandwidth=283 Hz/pixel and acquisition time=01:57 min.

At both anatomical locations, axial T2*-weighted structural images were acquired using a three-dimensional (3D) multiecho spoiled gradient echo sequence (Siemens Multi-Echo Data Image Combination (MEDIC)). In the upper cervical cord, the 20 axial–oblique slices were centred at the C2/C3 intervertebral disc, while in the lumbar cord they were centred at the widest point of the lumbar cord (lumbar enlargement) as appearing in the T2-weighted image.^19^ Four repetitions were acquired with the following parameters: slice thickness=2.5 mm, in-plane resolution=0.5×0.5 mm^2^, FOV=192×162 mm^2^, 5 echoes, TE of first echo=7 ms, echo spacing=3 ms, TR=44 ms, flip angle=11°, readout bandwidth=260 Hz/pixel, total acquisition time=7:16 min.

Diffusion-weighted images for diffusion tensor imaging (DTI) were acquired using a reduced FOV single-shot spin echo Echo Planar Imaging (EPI) sequence with identical slice prescription as the T2*-weighed images and consisting of 60 diffusion-weighted (b=500 s/mm^2^) and 7 T2-weighted (b=0 s/mm^2^) images. Acquisition parameters were: slice thickness=5 mm, in-plane resolution=0.76×0.76 mm^2^, FOV=133×30 mm^2^, TE=73 ms, TR=350 ms, readout bandwidth=768Hz/pixel, 5/8 partial Fourier in the phase-encoding direction. The acquisition was cardiac gated (3 slices per cycle, trigger delay=200 ms) and lasted approximately 8 min, depending on the heart rate.

In patients, an additional sagittal T2-weighted 2D turbo spin echo sequence of the lesion area was acquired with the following parameters: 20 slices, slice thickness=2.5 mm, in-plane resolution=0.3×0.3 mm^2^, FOV=220×200 mm^2^, TE=84 ms, TR=3500 ms, flip angle=160°, readout bandwidth=260 Hz/pixel, acquisition time=1:47 min.

### Processing of T2*-weighted images

An average of the four T2*-weighted structural images was created using serial longitudinal registration to account for within-scan motion,^20^ which was resliced to 5 mm slice thickness to increase signal-to-noise ratio. The resulting image was segmented for spinal cord using the semiautomatic 3D active surface method in JIM V7.0 software.^21^ Grey matter was segmented manually using subvoxel manual segmentation in JIM V7.0. White matter segmentation was obtained by subtracting grey matter from the spinal cord mask. Cross-sectional tissue areas including spinal cord area (SCA), grey matter area (GMA), and white matter area (WMA) were extracted from these segmentations and averaged across slices. In the lumbar enlargement, only three slices around the slice with the largest SCA were considered to ensure comparable and reproducible anatomical location.

### Processing of diffusion-weighted images

Diffusion-weighted images underwent artefact correction using the SPM-based ACID toolbox.^22^ All images were cropped to an in-plane FOV of 30×30 mm^2^ to exclude much of the non-spinal cord tissue. Eddy current and motion correction were applied using the Eddy Current and Motion Correction (ECMOCO) algorithm.^23^ The mean diffusion-weighted image was segmented for spinal cord using Propseg.^24^ DTI maps including fractional anisotropy (FA), axial diffusivity (AD), and radial diffusivity (RD) were obtained using the ACID robust-fitting algorithm.^22 25^ AD and RD represent the diffusivities parallel and perpendicular to the axons, respectively, under the assumption that the diffusion ellipsoid is aligned with the white matter tracts, which is usually the case in the spinal cord. FA is a measure of diffusion anisotropy, with a value of 0 representing isotropic and 1 representing full anisotropic diffusion. The mean diffusion-weighted image was spatially normalised to the PAM50 template using Spinal Cord Toolbox (V4.2.1), which shares the same coordinate system with the MNI-ICBM 152 template.^26^ The obtained deformation field was applied to all DTI maps. Mean values of FA, AD and RD were extracted within three white matter tracts of interest (dorsal, lateral and ventral columns) using the atlas-based analysis capabilities of Spinal Cord Toolbox.^27^ Partial volume effects were minimised by extracting the ‘maximum a posteriori’ value instead of the average within the tracts, where voxels at the edges are underrepresented.

### Clinical assessments

In patients, the neurological status and functional impairment were assessed according to the International Standards for Neurological Classification of Spinal Cord Injury (ISNCSCI) protocol.^28^ ISNCSCI assesses the strength of five key muscles in the upper and lower extremities on both sides on a scale of 0–5 (0: no motor function, 5: normal function) and the sensory function of 28 dermatome pairs in terms of light touch sensation and sharp–dull discrimination (pinprick) on a scale of 0–2 (0: no sensation/discrimination, 2: normal sensation/discrimination). Single scores summed up across all neurological levels are referred to as total motor, light touch and pinprick scores. In addition, Spinal Cord Independence Measure (SCIM) was used to assess daily life independence on a scale between 0 and 100.^29^

### Electrophysiological measurements

The electrophysiological examinations, including motor evoked potentials (MEP) for tibialis anterior and somatosensory evoked potentials (SEP) of the posterior tibialis nerve, were conducted according to the standard protocol of the European multicenter study about SCI (https://www.emsci.org/).

### Lesion segmentation

In patients, the lesion (hyperintense signal in the parenchyma) was segmented in the sagittal T2-weighted image. On the mid-sagittal slice, the following parameters were quantified as previously described^4^: mid-sagittal anterior–posterior lesion width (defined as the maximal anterior–posterior width of the lesion), mid-sagittal rostrocaudal lesion length (defined as the maximal rostrocaudal length of the lesion), mid-sagittal lesion area and mid-sagittal width of tissue bridges.

### Statistical analysis

Age and sex differences between patients and controls were assessed using Mann-Whitney U test and Fisher exact test, respectively. In the lumbar enlargement, we excluded six patients with SCI and one healthy control due to motion artefacts and/or signal dropout caused by orthopaedic implants. For assessing baseline macrostructural and microstructural differences, baseline SCA, GMA, WMA and DTI metrics within white matter tracts were compared using two-sample t-test (unpaired, one tailed, p<0.05). In addition, voxel-based analysis was performed in SPM 12, using a two-sample t-test in each voxel, where the statistical parametric maps were thresholded at p<0.005 (uncorrected), followed by a peak-level threshold of p<0.05 (family-wise error (FWE) corrected).

For longitudinal changes in SCA, GMA, WMA and DTI metrics, a linear mixed effect model was implemented using the nlme package in R (p<0.05). To investigate voxel-based longitudinal changes in the DTI maps, a similar linear mixed effect model with subject-varying intercept and rate of change (and allowing for interaction between groups) was implemented using the SwE toolbox.^30^ Voxels with p<0.05 (false discovery rate (FDR) corrected) at peak level were considered significant.

To compare relative atrophies of grey versus white matter, standardisation was applied on the patients’ GMA and WMA values. During standardisation, the average GMA (WMA) across controls was subtracted from the individual GMA (WMA), and the difference was divided by the SD of GMA (WMA) across controls. The resulting standardised score represents the number of SD by which the individual GMA (WMA) lies above or below the corresponding average value across all controls. A linear mixed effect model over time was applied on the individual differences between standardised GMA and WMA, where the intercept indicates baseline differences in the relative white and grey matter atrophy, and the slope indicates a linear change in relative atrophies over time.

We also investigated the ability of baseline (2 months post injury) MRI readouts to predict 1.5-year clinical scores, independently of the baseline clinical values. For this, a multiple regression model was applied in each voxel with the baseline clinical scores and voxel-wise baseline MRI readouts being the two predictors and the 1.5-year clinical score being the dependent variable. The initial cluster-defining threshold of p=0.005 was followed by a peak-level threshold of p<0.05 (FWE corrected).

## Results

### Patients’ characteristics and clinical outcomes

Of the 16 patients studied, 8 were tetraplegic and 8 paraplegic. Two patients were American Spinal Injury Association Impairment Scale (AIS) A (‘complete’), 1 AIS B (‘sensory incomplete’), 2 AIS C (‘motor incomplete’), and 11 AIS D (‘motor incomplete’) (table 1). For ISCNSCI and SCMI scores, a logarithmic fit with respect to time on the longitudinal values was superior to a linear fit, as shown by similar or lower p values of the slope, implying that recovery rate is faster soon after injury and decreases over time. Over 1.5 years post injury, patients recovered by 1.5 points per log month (95% CI: 0.6 to 2.3) on their ISNCSCI upper extremity motor score (p=0.002), and showed weak tendencies to improvement by 2.4 points (−0.8 to 5.6, p=0.131) on their lower extremity motor score and by 3.6 points (−0.8 to 8.0, p=0.101) on the light touch score. Pinprick scores did not change significantly over time (p=0.718). In the same period, SCIM score improved by 10.5 points per log month (3.5 to 17.5, p=0.005). There was no difference between patients and controls in terms of age (W=66, p=0.476). Electrophysiological characterisation demonstrated myelopathy in 13 of 16 patients, showing pathological tibial nerve SEP (3 normal, 6 delayed, 7 abolished) and/or pathological MEP recorded from tibialis anterior (motoneuron pool L4/5) and abductor hallucis muscle (motoneuron pool S1/S2) resulting in pathologic central motor conduction time (4 normal, 6 delayed, 6 abolished MEP). All motor complete paraplegic patients (AIS A–B) showed completely abolished SEP and MEP of the tibialis anterior muscles, whereas three patients with incomplete tetraplegia or paraplegia (AIS C–D) exhibited normal values.

### Lesion characteristics

Mid-sagittal tissue bridges were present in all motor incomplete patients (AIS C–D), with a mean (±SD) width of 4.9±2.3 mm at 2 months post injury, which showed no significant change over time (p=0.312). Of the 13 motor incomplete patients, 4 had no visible lesions, while the lesion could not be assessed in 4 patients due to metal artefacts. Motor complete patients (AIS A–B) did not have preserved mid-sagittal tissue bridges. In patients with visible lesions, the mean (±SD) mid-sagittal lesion width, length and area were 4.9±2.2 mm, 19.9±17.0 mm and 70.2±70.9 mm^2^, respectively, at 2 months post injury. The mid-sagittal lesion width decreased over time at a rate of 0.93 mm per month (95% CI: −0.16 to −0.02, p=0.016), while changes in lesion area and length were not significant.

### Longitudinal changes in cross-sectional tissue areas

At 2 months post injury, patients had smaller SCA (controls vs SCI: 85.4 vs 78.6 mm^2^, −7.9%, p=0.008) and WMA (72.8 vs 65.7 mm^2^, −9.7%, p=0.004) at C2/C3. In the lumbar enlargement, patients had smaller SCA (65.0 vs 56.1 mm^2^, −13.7%, p=0.003), WMA (45.4 vs 41.6 mm^2^, −8.5%, p=0.049) and GMA (19.6 vs 16.5 mm^2^, −15.9%, p=0.001).

In controls, none of the cross-sectional area metrics changed significantly over time. Compared with controls, patients had a significantly greater rate of change (negative direction, atrophy) for a number of metrics. At C2/C3, SCA decreased at a rate of 0.42% per month (0.33 mm^2^ per month, 95% CI: −0.47 to −0.20, controls vs SCI: p=0.002), GMA at 0.70% per month (0.09 mm^2^ per month, CI: −0.14 to −0.04, p=0.004), and WMA at 0.34% per month (0.23 mm^2^ per month, CI: −0.35 to −0.11, p=0.020) (figure 1C–E). In the lumbar enlargement, SCA decreased at a rate of 0.35% per month (0.21 mm^2^ per month, CI: −0.31 to −0.10, p<0.001) and WMA at 0.35% per month (0.15 mm^2^ per month CI: −0.25 to −0.05, p=0.007) (figure 1F–H).

### Longitudinal changes in DTI metrics

**Figure 2:**
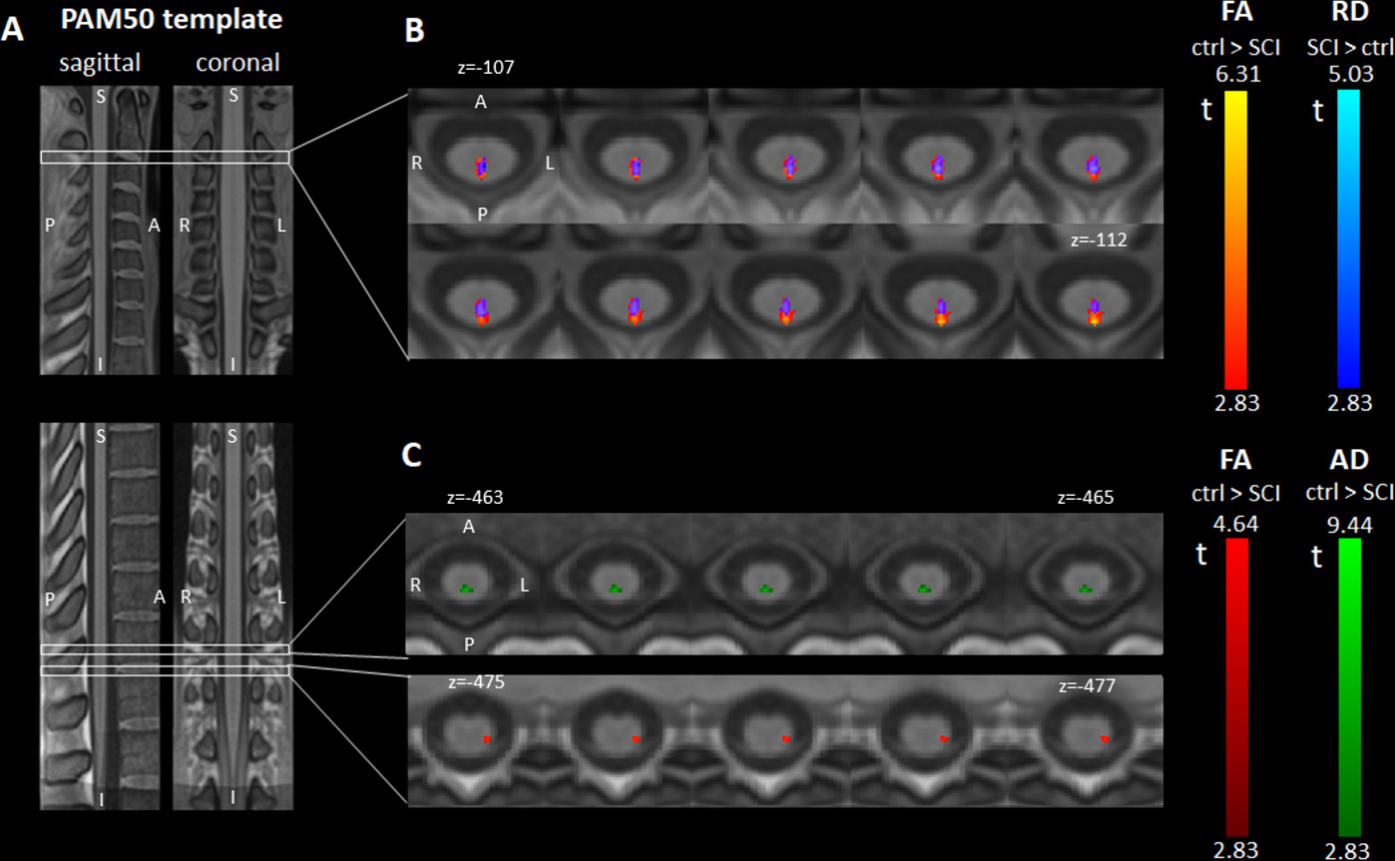
Differences in diffusion tensor imaging (DTI) metrics at 2 months post injury. (A) Sagittal and coronal views of the T1-weighted PAM50 spinal cord template used for the voxel-based and atlas-based DTI analyses. (B, C) Axial slices of parametric maps representing the t values of the voxel-based two-sample t-test comparing fractional anisotropy (FA) (displayed in red), radial diffusivity (RD) (blue) and axial diffusivity (AD) (green) between controls and patients with spinal cord injury (SCI). All parametric maps were created using SPM and are overlaid on the PAM50 template for display. The displayed clusters represent voxels with significant t values at an initial voxel-level threshold of 0.005 (uncorrected) followed by peak-level threshold of 0.05 (family-wise error corrected). (B) At C2/C3 (above the lesion), significant and overlapping clusters of FA decrease and RD increase were found in the dorsal column of patients with SCI. The overlapping region is displayed in violet. (C) In the lumbar enlargement (below the lesion), patients with SCI had lower FA in the left lateral column (corresponding to the left corticospinal tract) and lower AD in the dorsal column.

In patients, atlas-based analysis of DTI metrics revealed lower FA (controls vs SCI: 0.68 vs 0.60, −11.3%, p=0.008) and a trend to higher RD (0.63 vs 0.76 µm^2^/ms, +20.5%, p=0.059) in the dorsal column at C2/C3. In the lumbar enlargement, patients had lower FA in the lateral (0.54 vs 0.48, –10.3%, p=0.015) and ventral columns (0.48 vs 0.44, −9.7%, p=0.043) and lower AD in the dorsal column (2.11 vs 1.83 µm^2^/ms, −13.4%, p=0.005). Voxel-based analysis corroborated atlas-based findings: at C2/C3, dorsal column exhibited spatially specific FA decrease (PAM50 coordinates: x=0, y=−49, z=−106, z score=4.67, FWE-corrected p=0.002) and RD increase (x=0, y=−48, z=−109, z score=4.03, FWE-corrected p=0.019) (figure 2B). In the lumbar enlargement, the left lateral column had lower FA (x=−4, y=−47, z=−476, z score=3.75, FWE-corrected p=0.021) and the dorsal column had lower AD (x=0, y=−47, z=−462, z score=3.75, FWE-corrected p=0.014) (figure 2C).

**Figure 3:**
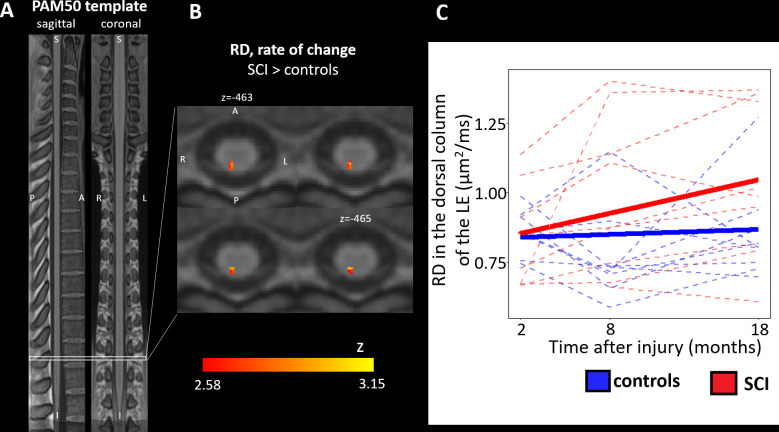
Longitudinal changes of diffusion tensor imaging (DTI) metrics. (A) Sagittal and coronal views of the T1-weighted PAM50 spinal cord template used for the voxel-based and atlas-based DTI analyses. (B) Axial slices of the parametric z map representing the z scores associated with the linear component of the voxel-based linear mixed effect model that models the evolution of radial diffusivity (RD) as a quadratic function over time. The displayed cluster represents voxels with significant t values at an initial voxel-level threshold of 0.005 (uncorrected) followed by peak-level threshold of 0.05 (false discovery rate corrected). In the dorsal column of patients with spinal cord injury (SCI), a significant cluster of voxels with RD increase was found in the lumbar enlargement. (C) Longitudinal changes in RD averaged within the dorsal column in the lumbar enlargement (below the injury). Dashed lines represent evolution in individual subjects, while the solid lines represent the quadratic fit in controls and patients with SCI separately. Patients with SCI showed a significant RD increase over 1.5 years after injury compared with controls.

Controls showed no significant changes over time in any of the three ROIs in the atlas-based, nor in the voxel-based analysis. In the dorsal column of the lumbar enlargement, patients showed a trend to increasing values for RD, at a rate of 1.2×10^−2^ µm^2^/ms per month (95% CI: 0.3×10^−3^ to 2.2×10^−2^ µm^2^/ms, controls vs SCI: p=0.097) (figure 3C), which was also confirmed in a voxel-based analysis (x=1, y=−47, z=−474, z score=3.15, FDR-corrected p=0.083) (figure 3B).

### Comparison between dynamic white and grey matter atrophy

**Figure 4:**
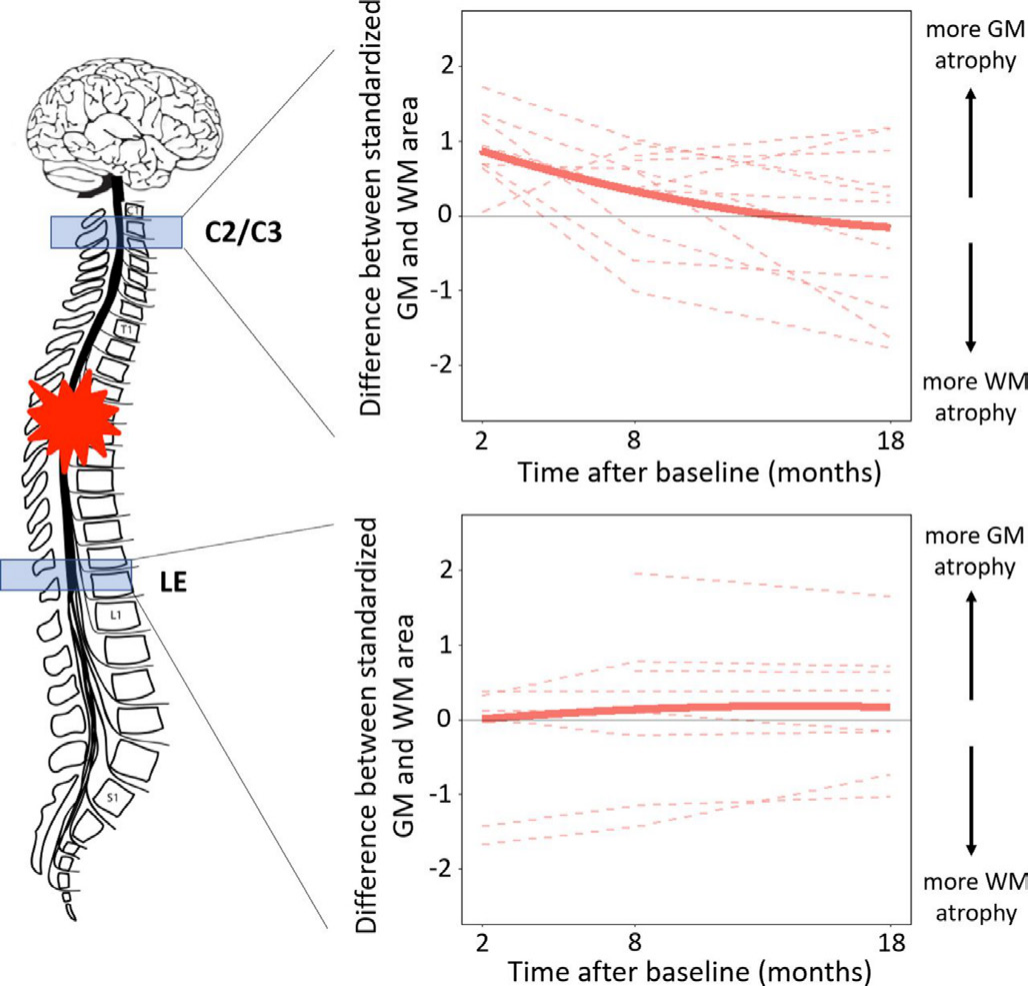
Longitudinal changes in the difference between spinal cord injury (SCI) patients' standardised white matter (WM) and grey matter (GM) areas over time at C2/C3 and in the lumbar enlargement (LE). For each patient with SCI, the standardised WM area is calculated by subtracting the average WM area in controls and dividing the result by the SD of WM area across controls (same approach for GM area). Dashed lines represent evolution in individual subjects, while the solid line represents the quadratic fit.

At C2/C3, patients with SCI had higher standardised GMA than standardised WMA by a score of 0.86 (p<0.001) at 2 months post-injury, but this difference declined over time at a rate of 0.064 per month (p=0.014), completely diminishing by 1.5 years post injury (figure 4). In the lumbar enlargement, standardised GMA and WMA values were not significantly different at any timepoint (figure 4).

### Relationship between baseline MRI measures and 1.5-year clinical scores

**Figure 5:**
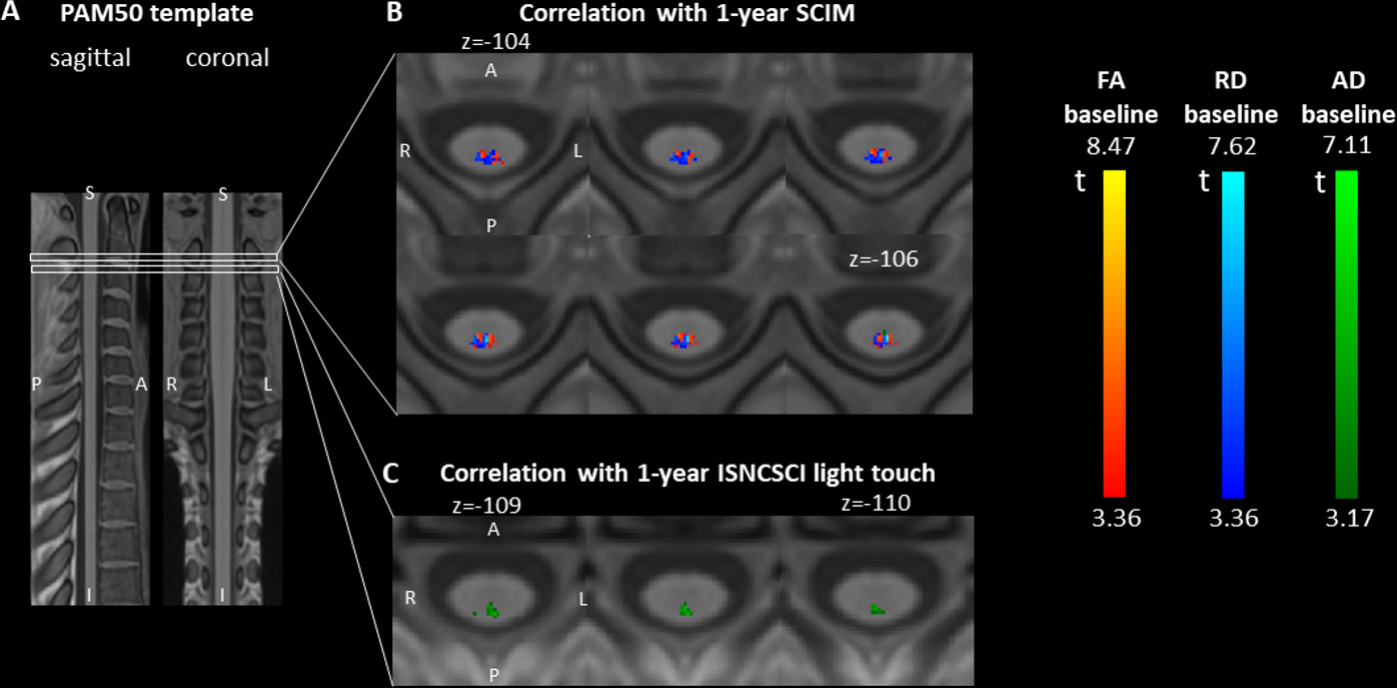
Associations between baseline diffusion tensor imaging (DTI) metrics and 1.5-year outcome. (A) Sagittal and coronal views of the T1-weighted PAM50 spinal cord template used for the voxel-based and atlas-based DTI analyses. (B, C) Axial slices of parametric t-maps representing the t values associated with the baseline DTI values in the prediction model. The displayed cluster represents voxels with significant t values at an initial voxel-level threshold of 0.005 (uncorrected) followed by a cluster-level extent threshold of 0.05 (false discovery rate corrected). The parametric map is overlaid on the T1-weighted PAM50 template. (B) In overlapping regions in the dorsal column at C2/C3, baseline fractional anisotropy (FA) and radial diffusivity (RD) in patients with spinal cord injury correlate with 1.5-year Spinal Cord Independence Measure (SCIM) score (positive correlation with FA, negative with RD), independent of baseline SCIM. (C) In the dorsal column at C2/C3, baseline axial diffusivity (AD) correlates positively with 1.5-year International Standards for Neurological Classification of Spinal Cord Injury (ISNCSCI) light touch score, independent of baseline light touch score.

In patients with SCI, baseline (2 months post injury) FA values in the dorsal column at C2/C3 correlated positively with the 1.5-year SCIM score, independently of the baseline SCIM score (x=−2, y=−47, z=−104, z score=4.18, FWE-corrected p=0.037). In overlapping regions, baseline RD correlated negatively with the 1.5-year SCIM score (x=−1, y=−47, z=−103, z score=4.01, FWE-corrected p=0.035) (figure 5B). At C2/C3, baseline AD values in the dorsal column correlated positively with the 1.5-year ISNCSCI light touch scores (x=−1, y=−49, z=−126, z score=4.15, p=0.026) (figure 5C). Baseline cross-sectional tissue areas did not correlate with 1.5-year outcome scores.

## Discussion

This study illustrates spatiotemporal trajectories of tissue-specific spinal cord neurodegeneration, both rostral and caudal to the level of injury. Importantly, it reveals differences in the evolution of tissue-specific changes below and above the level of injury. Baseline white matter diffusion metrics predicted clinical recovery.

### Early changes after SCI

As early as 2 months after injury, white matter atrophy was detectable both rostral and caudal to the injury. Although the pattern of anterograde and retrograde degeneration is different rostral and caudal to the injury, the magnitude of white matter atrophy was very similar at both levels. Rostral to injury (at C2/C3), the dorsal columns in patients exhibited lower FA driven by higher RD values, which has been associated with demyelination,^15 31 32^ but without similar findings in motor tracts. This finding suggests that rostral to injury anterograde degeneration of the sensory tracts is more prominent than retrograde degeneration of the motor tracts early after injury. Caudal to injury (in the lumbar enlargement), FA was lower in the lateral and ventral tracts, while AD was lower in the dorsal columns, providing evidence for both anterograde degeneration of motor tracts and retrograde degeneration of sensory tracts. Interestingly, the results suggest that white matter atrophy, both at C2/C3 and in the lumbar enlargement, occurs at the earliest timepoints after injury.

### Longitudinal changes after SCI

As in previous reports investigating spinal cord neurodegeneration,^8 10 18^ we observed a progressive remote white matter atrophy into the chronic stages of injury. Within 1.5 years after injury, white matter showed a consistent decline in tissue area, at a similar rate both at C2/C3 and in the lumbar enlargement (0.34% vs 0.35%), without significant signs of deceleration at 1.5 years post injury. At C2/C3, the observed spinal cord atrophy rate (grey and white matter combined) of 0.33 mm^2^ per month was in line with the previously reported value of 0.41 mm^2^ per month in SCI.^8^ In the lumbar enlargement, we did not find comparable values in the literature. Interestingly, the magnitude of white matter atrophy observed at 1.5 year post injury reached values seen in separate cohorts of chronic patients with SCI at C2/C3 (−14.9% vs −16.9%)^16^ and in the lumbar enlargement (−12.3% vs −10.8%),^17^ indicating decreasing atrophy rates after 1.5 years. Beside the progressive atrophy of white matter, the increase of RD in the lumbar enlargement over 1.5 years further supports the hypothesis that progressive neurodegeneration occurs over an extended time after injury, well into the chronic stage of injury.

While atrophy of the grey matter was only evident in the lumbar enlargement in the early stages of injury, GMA decreased within 1.5 years after injury, both rostral and caudal to the injury site. However, in contrast to white matter, we observed a discrepancy between findings at C2/C3 and in the lumbar enlargement. At C2/C3, grey matter showed no sign of atrophy 2 months post injury, but there was a steep decline afterwards, with GMA being already 14.4% smaller than aged-matched controls at 1.5 years post injury. In the lumbar enlargement, grey matter was considerably atrophied 2 months after injury, and exhibited only a moderate rate of progressive atrophy afterwards, which largely decelerated by 1.5 years post injury. The magnitude of grey matter atrophy in the lumbar enlargement reached at 1.5 years was similar to the value previously reported in chronic patients (−9.6% vs −13.0%),^17^ while it was still considerably lower at C2/C3 (−11.4% vs −30.0%),^16^ indicating continued grey matter atrophy beyond 1.5 years.

### Differential degenerative trajectories in grey and white matter

The discrepancy between C2/C3 and lumbar enlargement with respect to the onset of grey matter degeneration can be explained by the differential mechanisms involved in above-level and below-level grey matter degeneration. Grey matter atrophy above the lesion is thought to occur via synapses with interneurons in a process associated with the perturbation of propriospinal network,^33^ which is presumably a much slower process than the transsynaptic degeneration occurring in the grey matter below the lesion. Note that other mechanisms including changes in the amount and size of glial cells and vascular changes could also be involved in grey matter atrophy; however, their degree of contribution has not yet been established.^34^

### Prognostic value of early MRI findings

We showed that baseline MRI findings are associated with clinical recovery. Interestingly, we found that baseline DTI values are better predictors of clinical outcome than cross-sectional tissue areas, which can be explained by the large intrinsic variability and the unspecificity of tissue areas to microstructural changes. In particular, the degree of myelination in the dorsal column at C2/C3 (as revealed by RD) proved to be the best predictor for functional independence (ie, SCIM score), a key outcome score.

### Limitations

The study included a heterogeneous patient cohort in terms of injury type, severity and lesion level. Of the three patients with non-traumatic SCI, two suffered from an ischaemic injury and one from a haemorrhage due to a cavernoma. While functional recovery trajectories after traumatic and ischaemic injuries are rather similar,^35^ the impact of haemorrhage on degenerative changes and recovery profiles is understudied. The heterogeneity certainly impacts the generalisability of the results; for example, we expect to see more degeneration in the lumbar enlargement at the subacute stage in patients with a lower level of injury, and more atrophy in general in a pure AIS A cohort. While the sample size was rather small, we primarily focused on longitudinal analyses and the number of datapoints (68) provided sufficient power for the longitudinal statistics. A potential source of bias is the manual segmentation of grey matter and semiautomatic segmentation of the spinal cord. At present, automatic segmentation algorithms have been validated only for the cervical grey matter,^36^ while manual segmentation continues to remain the gold standard technique in the lumbar cord. To make manual segmentation as accurate and unbiased as possible, all segmentations were performed by the same experienced user (GD) in a blinded way. Therefore, we argue that the potential segmentation bias is systematic and does not affect the longitudinal results. Another limitation is the test–retest variability of the MRI readouts, which especially impacts the inherently noisier DTI metrics (figure 3). However, since the noise component is stochastic over data points, healthy controls did not show any longitudinal changes in any metric over time. This makes us confident that the statistically significant longitudinal changes in patients represent biologically meaningful degenerative processes.

### Conclusion

This study reveals distinct temporospatial dynamic trajectories of tissue-specific neurodegeneration above and below an SCI. It highlights that degeneration in the grey matter above the level of injury is initially lower than in the white matter but accelerates over time to reach similar magnitudes of atrophy by approximately 1.5 years post-SCI. Below the lesion, grey and white matter atrophy progress along similar trajectories. The clinical eloquence of these findings is reflected by the clinicopathological association between early degenerative changes and functional and neurological recovery. These MRI measures could be used to track the efficacy of therapeutic interventions, including rehabilitation.

## Data Availability

Data are available upon reasonable request. Anonymised grouped data will be shared by request from a qualified investigator.
